# Lower buprenorphine elimination rate constant is associated with lower opioid use

**DOI:** 10.1007/s00213-022-06202-9

**Published:** 2022-08-12

**Authors:** Hesham Farouk Elarabi, Amanda J. Lee, Abdu Adem, Abuelgasim Elrasheed, John Marsden, Hamad Al Ghaferi

**Affiliations:** 1 National Rehabilitation Centre, Abu Dhabi, United Arab Emirates; 2 Addictions Department, Division of Academic Psychiatry, Institute of Psychiatry, Psychology and Neuroscience, King’s College London, London, UK; 3 Medical Statistics Team, University of Aberdeen, Aberdeen, UK; 4 Faculty of Medicine, Khalifa University, Abu Dhabi, United Arab Emirates; 5 International Society of Addiction Medicine, Abu Dhabi, United Arab Emirates

**Keywords:** Associations, Buprenorphine elimination rate, Opioid use, Outcome prediction

## Abstract

**Background:**

Opioid craving is suggested to correlate with the rate of reduction in buprenorphine (BUP) plasma levels. No studies explored Buprenorphine elimination rate constant (BUP EL.R) as a predictor of opioid use or retention in BUP treatment.

**Methods:**

Analysis was performed using data from a randomized controlled trial of 141 adults with opioid use disorder (OUD) randomized to Incentivized Adherence and Abstinence monitoring (I-AAM; experimental (*n* = 70) and treatment-as-usual; control (*n* = 71). In the I-AAM, structured access to unsupervised BUP doses was provided up to 28 days contingent of adherence measured by Therapeutic Drug Monitoring and abstinence by Urinary Drug Screens (UDS). In contrast, the treatment-as-usual (control) provided unstructured access to unsupervised doses was provided for up to 14 days considering UDS results. The primary outcome was percentage negative UDS. The secondary outcome, retention in treatment, was continuous enrollment in the study and analysis was via intention-to-treat. Significant bivariate correlations with the outcomes were adjusted for group allocation.

**Results:**

A significant negative correlation between BUP EL.R and percentage negative opioid screens (Pearson correlation coefficient − 0.57, *p* < 0.01) was found. After adjusting for trial group, BUP EL.R was shown to be an independent predictor of percentage negative opioid screens (Standardized Beta Coefficient − 0.57, 95% CI − 221.57 to − 97.44, *R*^2^ 0.322).

**Conclusion:**

BUP EL.R predicted 32.2% of the variation in percentage negative opioid UDS and may serve as a potential promising tool in precision medicine of BUP treatment. Higher buprenorphine elimination is associated with higher positive opioid urine screens during treatment.

**Trial registration:**

ISRCTN41645723 retrospectively registered on 15/11/2015.

## Introduction

Opioid use disorder (OUD) is a global health and social burden that co-occurs with mental health disorders at a rate ranging from 45 to 55% (Davis et al. 2005). Medication-assisted treatment (MAT) using full opioid agonist such as methadone, and a partial opioid agonist such as buprenorphine (BUP) and BUP combination with naloxone is the first-line in recovery oriented care (WHO 2003). Response to MAT measured by reduction in opioid use and retention in treatment is subject to multiple factors including sociodemographic characteristics and clinical characteristics (Backmund et al. 2001; Poirier et al. 2003). The effect of these factors on treatment outcome however remains equivocal (Stuart et al. 2018).

Buprenorphine has a complex pharmacology with multiple factors affecting its elimination rate constant like absorption, distribution, and metabolism. BUP elimination rate constant follows a first-order kinetics (Fischer et al. 2015). BUP observes a low oral bioavailability of approximately 14% (Mendelson et al. 1997) and is extensively metabolized to Norbuprenorphine (N-BUP) which demonstrates 20% of the activity of BUP. In the liver, the major metabolic pathway for BUP is cytochrome 3A4 (CYP 3A4) (65%), followed by CYP 2C8 (30%). The remaining 5% of BUP is metabolized by CYP2C9, CYP2C18, and CYP2C19 isoforms. While limited information is available on the effect of the variability of activity level of the metabolic enzymes on BUP plasma levels, several studies show that potent CYP 3A4 inhibitors and inducers increase and reduce the BUP plasma levels, respectively (Elkader and Sproule 2005; Zhou et al. 2007). In contrast, there is an increasing body of knowledge to suggest the existence of large variability in the activity level of methadone metabolizing enzymes (CYP 2D6, Volpe et al. 2018). Such variability was demonstrated by higher adverse events in poor metabolizers and lower response in ultra-rapid methadone metabolizers (McCarthy et al. 2020).

Therefore, studying the factors associated with treatment outcomes of MAT in OUD may contribute to optimal treatment precision. This paper is a secondary analysis of a 16-week outpatient randomized clinical trial of 141 adults with OUD maintained on buprenorphine/naloxone film (BUP/NX-F) (STAR-T; Suboxone Treatment and Recovery–Trial) (Elarabi et al. 2021) and aims to examine the association of sociodemographic, clinical, and medication (i.e., BUP) related factors with both illicit opioid use and retention in treatment.

## Methods

### Participants

Included in the trial were adults aged 18 years and over with a current diagnosis of OUD who were voluntarily seeking treatment and residing in the United Arab Emirates (UAE) with evidence of stable accommodation. Patients were excluded if they reported benzodiazepine use in excess of 20 mg daily diazepam equivalent during the 28 days prior to admission, were known to have naloxone or BUP hypersensitivity, were pregnant or planning to get pregnant, showed signs of hepatic impairment (elevation of liver function tests three times normal), or reported a suicide attempt in the past 12 months. Adults with uncontrolled severe mental or physical illness judged to compromise safety, or patients with cognitive impairment (a score of less than 17 on the Mini Mental State Examination), or involvement in the criminal justice system with a likelihood of being arrested, were excluded (Elarabi et al. 2019, 2021).

### Setting

The present analyses were conducted for participants receiving inpatient and outpatient care at specialized substance use treatment facilities. Toxicological measures were performed at the clinical toxicology services at the National Rehabilitation Centre (NRC), which hold the status of a World Health Organisation Collaborating Centre, based in Abu Dhabi, United Arab Emirates.

### Primary and secondary outcomes

The primary outcome was opioid use demonstrated as percentage negative opioid UDS from total screens collected at each visit to the outpatient clinic. The secondary outcome was the number of participants completing the 16-week outpatient study period without interruption from the total number of participants included in the trial. Interruption in treatment was defined as missing three consecutive outpatient visits (Elarabi et al. 2021).

### Design

Opioid use was recorded according to results of Urinary Drug Screens (UDS) performed by a point-of-care test configured with a full panel of opiates/opioids and confirmed by gas chromatography tandem mass spectrometry. In contrast, liquid chromatography tandem mass spectrometry was used to detect and quantify BUP in blood.

### Measures

Co-occurring mental health disorders were screened using the Patient Health Questionnaire (PHQ-9) (Kroenke et al. 2001) for depression; Generalized Anxiety Disorder (GAD-7) (Spitzer et al. 2006); for anxiety; Barratt Impulsiveness Scale (BIS-11) (Patton et al. 1995) for impulsiveness; Personality Disorder Screener (PDS) (Kessler et al. 1998) for personality disorders; Addiction Severity Index Lite version (ASI-Lite) (Cacciola et al. 2007) for the seven domains addiction severity; Work and Social Adjustment Scale (WSAS) (Mundt et al. 2002) for social and work impairment; and the Pittsburgh Sleep Quality Index (PSQI) (Buysse et al. 1989) for quality of sleep.

### Study procedures

Baseline sociodemographic data were recorded at admission to the inpatient care during which induction and stabilization on BUP/NX was performed. The total 24-h BUP/NX-F dose required to maintain a Clinical Opioid Withdrawal Score (COWS, Wesson and Ling 2003) score of ≤ 5 was set as the BUP/NX-F daily stabilization dose. Measures for co-occurring disorders and addiction severity were administered during the stabilization phase.

At steady state concentration (assumed after 2 weeks of unchanged BUP/NX-F dose), three blood samples were drawn from all participants. The first sample was drawn 40 min post the BUP dose representing the peak plasma concentration; the second sample was drawn 30 min prior to the BUP/NX-F dose representing trough plasma concentration and repeated 48 h later. Applying a pharmacokinetic model, the measured peak and trough BUP concentrations at steady-state concentration were used to estimate the BUP EL.R constant (Elarabi et al. 2020) during the inpatient treatment. Quantitation of BUP was done using liquid chromatography tandem spectrometry according validated method that was tested for clinical feasibility and accuracy. A fourth blood sample was drawn at random and BUP plasma level was quantified to validate the estimated BUP elimination rate during the pilot phase to demonstrate the feasibility and reliability of therapeutic drug monitoring (TDM) and pharmacokinetic model adopted in this trial (Elarabi et al. 2020).

At the end of the inpatient phase, participants were randomized to receive 16-weeks of BUP/NX-F and Incentivized Adherence and Abstinence Monitoring (I-AAM; experimental group, *n* = 70), and BUP/NX-F treatment-as-usual (control group, *n* = 71). Participants were required to have 12 UDS notwithstanding the protocol in each trial group. The I-AAM intervention applied contingency management (CM) using BUP/NX-F take-home doses (unsupervised prescription) of up-to 4-weeks as the incentive contingent of abstinence measured by UDS, and adherence determined by TDM. STAR-T introduced a novel application of TDM to monitor adherence via measuring, predicting and interpreting BUP plasma levels (Elarabi et al. 2020). In contrast, participants allocated to the TAU received take-home doses up to 2 weeks contingent of abstinence accessed by UDS (Elarabi et al. 2021).

During the outpatient phase, fine dose adjustments for BUP/NX-F were guided using multi-factorial assessment (Elarabi et al. 2019). These factors detailed in the study manual found on open science frame work (https://osf.io/jsa2y/) included mood and functional assessments of the participant, relapse prevention and management skills, participant’s overall performance, and craving assessed by both Minnesota Cocaine Craving Scale adapted for opioids (Halikas et al. 1991) and pupil reflexes captured using a hand held pupilometer (Fehringer 2021). Blood samples were collected in the experimental group to measure BUP concentration and evaluate adherence with BUP/NX-F. Adherence was assumed if the difference between the measured BUP and the predicted BUP concentration using the pharmacokinetic model was within 20% (Fischer et al. 2015; Elarabi et al. 2021). UDS were performed on each visit to the outpatient clinic according to the schedule set in each arm. The number of UDS performed in both study groups were examined for significance (Elarabi et al. 2021).

### Statistical analyses

Analyses was performed for the *total sample* (*n* = 141) according to intention-to-treat (ITT). Bivariate correlations (Pearson or Spearman’s rho) were applied to explore the linear association of sociodemographic characteristics, and clinical characteristics with percentage negative opioid screens and retention in treatment. Variables showing significant bivariate association were then examined for independence by linear regression after adjusting for the randomization groups. The software used was Statistical Package for Social Sciences version 24.0 with a two-sided *p* value of < 0.05 to determine statistical significance.

## Results

The primary outcome set for clinical trial was percentage negative opioid use was significantly higher in the I-AAM compared to the TAU group [(76.71% (SD 25.0%) versus 63.4% (SD 34.7%)] with a mean difference of 13.3% (95% CI 3.2 to 23.3; *d* = 0.44; 95% CI 0.10 to 0.87). In contrast, the secondary outcome, retention in treatment, defined as *completion* of the 16-week study period without interruption, was not statistically significantly different between the I-AAM group [(*n* = 40; 57.1%) and the TAU group (*n* = 33, 46.4%), odds ratio 1.54, 95% CI 0.79 to 2.98] (Elarabi et al. 2021). There was no difference in the mean number of UDS performed in the study groups [I-AAM 14.0 (SD 7.89) versus TAU 11.93 (SD 7.93), 95% CI -0.55 to 4.69].

### Participant characteristics

Table 1 summarizes the baseline characteristics of 141 participants stabilized on BUP/NX-F. Almost all participants were males (98.6%) with a mean age of 29.9 years (SD 8.16).

**Table 1:** Characteristics of participants at baseline

Variable	Total participants (*n* = 141)
Male	139 (98.6)
Age at first use	17.74 (4.13)
Age at presentation	29.0 (8.16)
Duration of illness	11.4 (7.60)
Family history of substance use	21 (14.9)
Heroin/Morphine Injecting use	67 (47.5)
Heroin/Morphine non-injecting use	43 (30.5)
Tramadol	31 (22.0)
Patient Health Questionaire-9	13.2 (6.72)
Generalized Anxiety Disorder-7	10 (5–17)
Pittsburgh Sleep Quality Index	10.2 (4.98)
Barratt Impulsiveness Scale-11	71.7 (14.6)
Work and Social Adjustability Scale	23.12 (14.6)
Addiction Severity Index-Medical	0.00 (0.00–0.35)
Addiction Severity Index-Social	0.50 (0.25–0.75)
Addiction Severity Index-Alcohol	0.00 (0.00–0.10)
Addiction Severity Index-Drug	0.22 (0.10–0.36)
Addiction Severity Index-Legal	0.00 (0.00–0.20)
Addiction Severity Index–Family	0.20 (0.04–0.48)
Addiction Severity Index-Mental	0.47 (0.12–0.70)
Border line Personality Disorder	86 (81.9)
Obsessive Compulsive Personality Disorder	29 (28.7)
Avoidant Personality Disorder	83 (80.6)
Dependent Personality Disorder	57 (60.0)
Anxious Personality Disorder	64 (62.1)
Paranoid Personality Disorder	43 (30.5)
Buprenorphine dose (mg/day)	14.8 (4.0)
Buprenorphine Elimination Rate constant (ng mL/h − ^1^)	0.047 (0.03–0.09)
Buprenorphine trough concentration (ng/mL)	1.33 (0.55–22.56)

Data are presented as mean (standard deviation), median (Interquartile range), number (percentage)

The mean dose of buprenorphine/naloxone for all randomized participants was 14.8 mg (SD 4.0) with a dose range of 16 (8 mg to 24 mg). Specifically, the mean dose for the participants completing 4 weeks was 15.2 mg (SD 4.1), and 15.4 (SD 4.0) for participants completing 8 and 12 weeks, and 15.48 (SD 4.0) for participants completing the 16-week trial without interruption.

### Association with percentage of negative opioid screens and retention in treatment

Table 2 summarizes the bivariate correlations of participant characteristics with percentage negative opioid screens and retention in treatment. A positive correlation with percentage negative opioid use suggests lower opioid use. For example, the older the participant, the higher the percentage negative opioid screens or the lower likelihood of using opioids. In contrast, a negative correlation suggests that increase in scores is associated with lower percentage negative opioid use or higher opioid use. For example, participants with higher scores on clinical scales like Patient Health Questionnaire and Addiction Severity Index-Drug Domain suggesting higher severity in depression and addiction severity index are more likely to use illicit opioid use. In contrast, a positive relation with retention (completion of 16 weeks follow up) suggests that participants with older age longer duration of illness and higher scores on clinical scales are more likely to complete the study period or observed higher retention rate. In contrast, a negative association suggests that participants are likely to observe lower retention rate.

**Table 2:** Correlation coefficients of participant characteristics with percentage negative opioid screens and retention in treatment

Variable	Correlation coefficient with percentage negative opioid screens^a^	95% confident interval	Correlation coefficient with retention^b^	95% confidence interval
Age at first use	0.100	− 0.072 to 0.265	0.115	− 0.056 to 0.279
Age	0.17	0.04 to 0.334	0.008	− 0.162 to 0.178
Duration of illness	0.133	− 0.033 to 0.292	− 0.011	− 0.181 to 0.159
Participants living Abu Dhabi^c^	Spearman’s rho − 0.140	− 0.303 to 0.031	0.038	− 0.133 to 0.207
Family history of substance use	Spearman’s rho 0.03	− 0.35 to 0.294	0.042	− 0.131 to 0.213
Primary type and injecting opioid	Spearman’s rho 0.11	− 0.109 to 0.221	− 0.099	− 0.265 to 0.072
Patient Health Questionnaire-9 items	− 0.072	− 0.243 to 0.104	− 0.169	− 0.338 to 0.011
Generalized Anxiety Disorder-7 items	0.06	− 0.126 to 0.216	0.062	− 0.116 to 0.236
Pittsburgh Sleep Quality Index	0.176	− 0.11 to 0.351	− 0.035	− 0.225 to 0.158
Barratt Impulsiveness Scale-11 items	− 0.034	− 0.335 to 0.160	− 0.063	− 0.258 to 0.137
Work and Social Adjustability Scale	− 0.03	− 0.334 to 0.062	− 0.055	− 0.259 to 0.154
Addiction Severity Index-Medical	0.108	− 0.06 to 0.268	0.114	− 0.058 to 0.278
Addiction Severity Index-Social	0.071	− 0.11 to 0.246	− 0.023	− 0.225 to 0.158
Addiction Severity Index-Alcohol	0.016	− 0.163 to 0.194	− 0.008	− 0.191 to 0.176
Addiction Severity Index-Legal	0.07	− 0.172 to 0.185	0.021	− 0.164 to 0.204
Addiction Severity Index-Drug	− 0.126	− 0.296 to 0.051	− 0.138	− 0.312 to 0.044
Addiction Severity Index-Family	0.01	− 0.156 to 0.174	0.118	− 0.053 to 0.283
Addiction Severity Index-Mental Health	0.105	− 0.062 to 0.265	0.021	− 0.164 to 0.204
Borderline Personality Disorder	Spearman’s rho	− 0.203 to 0.180	0.152	− 0.046 to 0.339
Obsessive Compulsive Personality	− 0.12	− 0.192 to 0.199	0.028	− 0.174 to 0.228
Antisocial Personality Disorder	Spearman’s rho 0.05	− 0.205 to 0.189	0.082	− 0.119 to 0.277
Dependent Personality Disorder	Spearman’s rho 0.09	− 0.189 to 0.214	0.123	− 0.087 to 0.322
Anxious Personality Disorder	Spearman’s rho 0.12	− 0.205 to 0.182	0.014	− 0.186 to 0.212
Paranoid Personality Disorder	Spearman’s rho 0.03	− 0.248 to 0.146	0.128	− 0.078 to 0.322
Buprenorphine dose	Spearman’s rho 0.07	− 0.005 to 0.320	0.161	− 0.011 to 0.324
Buprenorphine trough concentration	0.162	− 0.186 to 0.245	0.001	− 0.267 to 0.269
Buprenorphine Elimination Rate constant	0.031 − 0.567	− 0.720 to − 0.361	− 0.092	− 0.349 to 0.178

^a^Pearson correlation coefficient are shown unless otherwise stated

^b^Spearman correlation coefficient

^c^Participants who live in Abu Dhabi metropolitan demonstrated a negative correlation with percentage negative opioid screens or higher use of opioids compared to those living outside the city of Abu Dhabi

#### Associations with sociodemographic characteristics

Percentage negative opioid screens increases with older age [Pearson correlation coefficient = 0.17, 95% confident interval (CI) 0.04 to 0.33], i.e., in this analysis, older adults used less illicit opioids compared to younger adults. However, the statistical significance of this relationship diminished upon adjusting for trial group (Standardized Beta Coefficient 0.60, 95% CI − 0.007 to 1.21). No statistically significant correlation was found with any other sociodemographic factor and percentage negative opioid use nor retention in treatment.

#### Associations with co-occurring mental health disorders and domains of addiction severity

No statistically significant correlation was found between measures of depression, anxiety, impulsiveness, sleep disorders, personality disorders, addiction severity with wither percentage negative opioid use and retention in treatment.

#### Associations with buprenorphine plasma concentration and buprenorphine daily dose

No statistically significant correlation was found between the mean trough BUP plasma concentration or BUP/NX-F daily dose with either treatment outcomes (percentage negative opioid use and retention in treatment).

#### Associations with buprenorphine elimination rate constant

A significant negative correlation between BUP EL.R and percentage negative opioid screens (Pearson correlation coefficient − 0.570, 95% CI − 0.720 to − 0.361) was found. After adjusting for trial group, BUP EL.R was shown to be an independent predictor of actual percentage negative opioid screens (Standardized Beta Coefficient − 0.567, 95% CI − 221.57 to − 97.44, R^2^ 0.322). In contrast, no significant correlation was found between BUP EL.R and retention in treatment (Spearman’s rho − 0.092, 95% CI − 0.349 to 0.178).

**Fig. 1:**
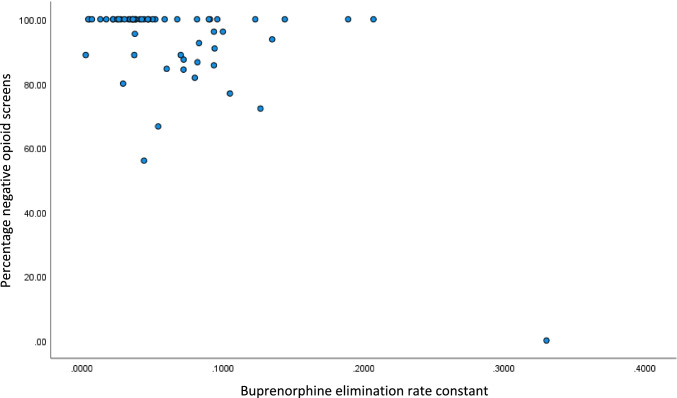
Scatterplot of buprenorphine elimination rate constant and percentage negative opioid screens

Figure 1 shows the relationship between BUP EL.R and percentage opioid negative screens. There is no obvious polynomial relationship. However, the scatterplot suggest some evidence of subgroups within the sample; one group showing a high percentage opioid negative screens irrespective of BUP EL.R and another showing decreasing percent opioid negative screens as BUP EL.R increases.

## Discussion

The objective of this analysis was to explore the association of sociodemographic, clinical, and BUP related characteristics with opioid use and retention in BUP based treatment of OUD. Results suggest that rapid BUP elimination rate is associated with an increase in urine screens positive for opioids in adults receiving BUP treatment. In the present analysis, two possible subgroups exist, one showing a high percentage opioid negative screens irrespective of BUP EL.R and another showing decreasing percent opioid negative screens as BUP EL.R increases. This may be due to the medium size association between BUP EL.R and opioid use and that BUP EL.R explained opioid use in almost one-third of the participants. It is important to note that multiple biological, psychological, social, and spiritual factors contribute to the recovery process and is not limited to a single biomarker. Clinically, applying a prediction response model to BUP treatment possibly contribute to enhancing the precision of BUP maintenance treatment which in turn leads to enhanced effectiveness, cost-effectives, and expanding access to MAT using BUP. To the authors’ best knowledge, BUP EL.R prediction of opioid use has not been previously studied.

A possible explanation for the association of BUP EL.R with opioid use is that a rapid fall in BUP blood levels may predispose withdrawal and/or craving hence increasing the tendency to use opioids. This seems to be consistent with a hypothesis from a previous study suggesting that the rate of reduction in BUP plasma levels rather than absolute BUP blood concentration is directly association with opioid craving (Lopatko et al. 2003). BUP elimination follows a first-order kinetics (Fischer et al. 2015) with 30% excreted by the kidney as a glucourinate conjugate. BUP plasma levels is affected by multiple factors including the activity level of the metabolic pathways as BUP is 100% metabolized in the liver predominately by CYP 3A4. Limited information exists on the impact of variability in the activity of the metabolic enzymes on BUP treatment outcomes. Nevertheless, reduction and rise in BUP plasma levels was established with CYP 3A4 inducer and inhibitor drugs (Elkader and Sproule 2005; Zhou et al. 2007). Moreover, BUP was reported to observe a drug-food interaction with significant differences reported for BUP plasma concentrations before and after food (Elkader and Sproule 2005).

The present analysis shows a positive association, although not statistically significant, between mean maintenance daily dose of BUP/NX-F with opioid use nor with retention in treatment. In contrast, previous studies showed that higher BUP daily dose range, i.e., 12 mg to 24 mg, is associated with higher reduction opioid use during maintenance treatment compared to a daily dose range of 8 mg to 16 mg (Mattick et al. 2003). Furthermore, results from a secondary analysis of adults with OUD receiving BUP and methadone (Hser et al. 2014) support that BUP daily doses over 16 mg and methadone daily doses above 60 mg were significantly associated with higher retention in treatment over 24 weeks (Hser et al. 2014). Nevertheless, a recent systematic review found no conclusive evidence on the association of BUP daily dose with treatment outcomes (Hjelmström et al. 2020). The systematic review concluded adjusting BUP maintenance dose according to clinical response, i.e., opioid use, may be optimal compared to dose adjustment in response to signs and symptoms of withdrawal and craving (Hjelmström et al. 2020). This may suggest a difference in the BUP doses required to suppress craving compared to doses required to suppress opioid use over time.

In contrast, adequate daily dosing of methadone was associated with lower opioid use and higher retention in treatment (Darke et al. 2007). Variability in the genotypes of the methadone metabolizing enzyme CYP 2D6 is reported among multiple factors that influence methadone daily dose (Fonseca et al. 2011). For example, individuals with ultra-rapid metabolic activity of CYP 2D6 observe sub-therapeutic methadone levels clinically described as “*deficient patient satisfaction*” (Pérez de los Cobos 2007) and “*poor*” performance on methadone with “*frequent withdrawal symptoms*” (Haile et al. 2008). In response to variability in the activity level of the metabolizing enzymes, personalizing methadone doses in patients with ultra-rapid, intermediate, and ultra-slow activity has been suggested (McCarthy et al. 2020). In fact, some practice guidelines for methadone maintenance (College of Physicians and Surgeons of British Columbia 2015) recommended a split dose every 12 h for ultra-rapid metabolizers. Similar to methadone, inter-individual variability in patient characteristics results in variable BUP maintenance doses (Hillhouse et al. 2011).

Results from the present analysis suggest that BUP plasma concentrations are not significantly associated with opioid use. Imaging and biochemical studies suggest that a minimum BUP concentration of 1 ng/mL corresponding to 50% MOR occupancy was required to control opioid withdrawal symptoms and craving (Greenwald et al. 2003). BUP levels of 1 to 3 ng/mL were reported to be optimal for reduction in opioid withdrawal symptoms, reduction in craving, and reduction in illicit opioid use (Hiemke et al. 2017). In the present analysis, other than age, no statistically significant association was established between the sociodemographic characteristics and opioid use, or with retention in BUP treatment. However, the association showing that older adults compared to younger adults are less likely to use opioids lost statistical significance upon adjusting for randomization.

Findings from the present analysis showing lack of association of treatment outcomes with sociodemographic characteristics are congruent with previous studies examining the association of sociodemographic characteristics with opioid use (Backmund et al. 2001; Saxon et al. 1996). In contrast, previous studies suggested that older compared to younger adults are more likely to demonstrate higher retention in opioid treatment and lower use of illicit opioids during MAT (Dreifuss et al. 2013; Weiss et al. 2011; Hser et al. 2014). Duration of illness was also reported to be associated with illicit opioid use during MAT (Dreifuss et al. 2013). Laqueille and colleagues (2001) reported that individuals with duration of illness under 10 years are less likely to use opioids compared to individuals with duration of illness above 10 years MAT. Finally, in the present study, injecting drug use was not associated with retention rates unlike data suggesting the association of baseline injecting drug use with higher treatment attrition (Dayal and Balhara 2017).

The present exploratory findings are based on the analysis of a relatively small sample; the majority of whom were male. The authors strongly encourage that future studies examine the present findings in a larger sample and also explore factors that may influence BUP EL.R.

## Conclusion

The likelihood of using illicit opioids during buprenorphine treatment increases with higher buprenorphine elimination rate. Predicting response to buprenorphine using buprenorphine elimination rate constant may contribute to the precision of buprenorphine based treatment. Further studies with larger samples including females are required to support the exploratory results and examine the impact of adjusting buprenorphine dose size and frequency according to buprenorphine elimination rate constant. Efforts on developing clinical algorithms integrating these findings in clinical practice are strongly encouraged.
